# Prognostic value of the derived inflammatory marker SIRI in postmenopausal women with coronary artery disease

**DOI:** 10.3389/fcvm.2024.1418781

**Published:** 2024-12-20

**Authors:** Pengli Yang, Rui Xue, Yuhang Wei, Chenxi Cao, Songcheng Yu, Shanling Peng, Wenjing Zhang, Yunzhe Wang, Yingying Zheng, Gangqiong Liu

**Affiliations:** ^1^ Department of Cardiology, Key Laboratory of Cardiac Injury and Repair of Henan Province, First Affiliated Hospital of Zhengzhou University, Zhengzhou, China; ^2^ Medical Research Center, The First Affiliated Hospital of Zhengzhou University, Zhengzhou University, Zhengzhou, Henan, China; ^3^ College of Public Health, Zhengzhou University, Zhengzhou, China; ^4^ Department of Emergency, The First Affiliated Hospital of Zhengzhou University, Zhengzhou University, Zhengzhou, Henan, China

**Keywords:** systemic inflammatory response index, postmenopausal women, coronary artery disease, adverse prognosis, derived inflammatory marker

## Abstract

**Objective:**

The aim of this study was to explore the predictive value of the Systemic Inflammatory Response Index (SIRI) for the prognosis of older postmenopausal women with coronary artery disease (CAD).

**Patients and methods:**

This retrospective cohort study included 617 postmenopausal female patients aged 50 years or older with a CAD diagnosis confirmed by coronary angiography seen at the First Affiliated Hospital of Zhengzhou University from January 2019 to December 2020. Patients were divided into three groups based on SIRI tertiles. Primary endpoints were all-cause mortality (ACM) and cardiac mortality (CM), and secondary endpoints were major adverse cardiovascular events (MACEs) and major adverse cardiovascular and cerebrovascular events (MACCEs).

**Results:**

The frequencies of all adverse outcomes were greater in the high level (third tertile) SIRI group than in the low level (first tertile) SIRI group. Multivariable regression analysis showed that compared to the low level SIRI group, the high level SIRI group had a 1.581-fold greater risk of ACM [hazard ratio (HR) = 2.581, 95% confidence interval (CI): 1.045–6.373, *p* = 0.040) and a 1.798-fold greater risk of CM (HR = 2.798, 95% CI: 0.972–8.060, *p* = 0.057). In addition, the risks of MACEs and MACCEs were 62.3% (HR = 1.623, 95% CI: 1.123–2.346, *p* = 0.01) and 55.8% (HR = 1.558, 95% CI: 1.100–2.207, *p* = 0.012) greater in the high level SIRI group compared with the low level SIRI group. Kaplan–Meier survival analyses confirmed that the high SIRI level was associated with increased risks of ACM (*p* = 0.001), CM (*p* = 0.005), MACEs (*p* = 0.003), and MACCEs (*p* = 0.005).

**Conclusion:**

This retrospective study demonstrates that the novel derived inflammatory index SIRI can effectively predict the risk of multiple adverse outcomes in postmenopausal women with CAD.

## Introduction

Coronary artery disease (CAD) is a form of ischemic cardiomyopathy in which the development of atherosclerosis leads to coronary stenosis. In the Asian population, which accounts for nearly half of the world's population, the prevalence of CAD has increased dramatically with the changes in dietary structure resulting from rapid economic development (1). Although men have a greater risk of developing coronary heart disease overall, CAD remains the leading cause of death among women (2). Moreover, the protective effect of estrogen is a main factor contributing to the reduced risk of coronary heart disease of women compared with men (3), and after menopause, the incidence of coronary heart disease in women is comparable to that in men (4). Women with CAD have a worse prognosis than men with CAD, primarily because women are more likely to experience non-severely obstructive CAD and atypical symptoms (5–8). Accordingly, reliable markers for predicting poor prognosis among post-menopausal women with CAD are urgently needed.

Atherosclerosis encompasses both localized and systemic chronic inflammation triggered by lipid accumulation. Localized inflammation induces the migration and aggregation of peripheral blood immune cells into the subendothelium through activation of vascular endothelial cells and their high expression of chemotactic adhesion molecules (9). The classical inflammatory marker C reactive protein (CRP) was shown to have independent predictive value in male and female patients with CAD, patients with acute coronary syndrome (ACS) and patients with chronic coronary syndrome (CCS) (10–12). The peripheral blood leukocyte level also has been shown to be strongly and independently associated with CAD, and leukocyte count has been correlated with the diagnosis and severity of CAD as well as with poor prognosis in patients with different types of CAD (13). Specific correlations have been reported between elevated eosinophil, monocyte, and neutrophil counts among peripheral blood leukocyte counts and an increased risk of CAD (14). A novel derived inflammatory marker termed the Systemic Inflammatory Response Index (SIRI) has been established as a comprehensive inflammatory indicator that reflects the relative levels and balance of monocytes, neutrophils, and lymphocytes. SIRI was first defined to predict cancer prognosis (15), and further studies have demonstrated its good predictive value for prognosis in patients with coronary heart disease (16, 17). However, to our knowledge, no studies have explored the predictive value of the SIRI for the prognosis of postmenopausal women with CAD.

The present study aimed to investigate the predictive value of the SIRI for the occurrence of death and adverse cardiovascular and cerebrovascular events in older postmenopausal women with CAD. The results of this study may provide an experimental basis for the clinical application of the SIRI as a valuable biomarker in these patients.

## Methods

### Experimental design and study population

This study was a single-center, retrospective, observational cohort study and included 672 older postmenopausal female patients who were diagnosed with CAD by coronary angiography at the First Affiliated Hospital of Zhengzhou University from January 2019 to December 2020. Coronary angiography and qualitative and quantitative coronary arteriographic analyses were performed by experienced interventional cardiologists. Patients with no contraindications were treated with oral antiplatelet agents and statins after diagnosis. A complete clinical history was available for all included patients.

The inclusion criteria for this study were: (1) age ≥50 years, (2) female sex and postmenopausal status; (3) confirmed diagnosis of CAD based on coronary angiography showing stenosis ≥50% in at least one vessel; and (4) complete clinical profile with no missing data. ACS included all cases of unstable angina, ST-elevation myocardial infarction, and non-ST-segment elevation myocardial infarction, whereas CCS was defined as stable angina. The exclusion criteria for this study included the presence of: (1) any acute infectious disease, (2) any tumor type (unless the tumor had been surgically removed or cured), (3) any autoimmune or hematologic disease, and (4) severe heart failure, heart valve disease, structural heart disease, or cardiomyopathy.

The study design and procedures complied with the tenets of the Declaration of Helsinki, and the protocol was approved by the Ethics Committee of the First Affiliated Hospital of Zhengzhou University. Follow-up data were obtained by reviewing medical records or via telephone interviews.

### Definition of SIRI

The novel derived inflammatory index SIRI selected for evaluation in this article is an important indicator of the state of systemic inflammation (15). The SIRI is calculated by multiplying the absolute neutrophil value by the absolute monocyte value divided by the absolute lymphocyte value.

### Clinical testing

Venous blood samples from patients who had been fasting for at least 8 h were collected within 24 h of admission for laboratory testing. Medical history data for each patient, including age, history of diabetes, history of hypertension, and history of atrial fibrillation, were obtained from admission charts. Laboratory results were recorded, including those of routine blood tests (absolute leukocyte count, absolute neutrophil count, absolute monocyte count, and platelet count); lipid levels (total cholesterol, triglycerides, low-density lipoprotein cholesterol [LDL-C], and high-density lipoprotein cholesterol [HDL-C]); fasting glucose and glycosylated hemoglobin; hepatic and renal functional parameters [creatinine, urea, uric acid, alanine transaminase (ALT), aspartate transaminase (AST), total bilirubin, direct bilirubin, indirect bilirubin, and albumin]; and blood coagulation markers (fibrinogen and D-dimer). The number of coronary artery lesions, lesion severity, and the presence or absence of left main artery lesions were recorded according to the patient's coronary angiography findings.

### Clinical endpoints and follow-up

We analyzed both primary and secondary endpoints. The primary endpoint was long-term mortality, including all-cause mortality (ACM) and cardiac mortality (CM). The secondary endpoints included major adverse cardiac events (MACEs) and major adverse cardiovascular and cerebrovascular events (MACCEs). ACM was defined as patient death from any cause, and CM was defined as patient death from a cardiac cause, including heart failure, myocardial infarction, or malignant arrhythmia, and other unexplained deaths for which non-cardiac causes could be definitively excluded. MACEs included ACM, nonfatal myocardial infarction, acute heart failure, target vessel revascularization (percutaneous coronary intervention or coronary artery bypass graft therapy), new malignant arrhythmia on discharge (transient or continuous ventricular tachycardia, ventricular fibrillation, atrial fibrillation, atrial flutter, and second- to third-degree atrioventricular block), and angina pectoris that required hospitalization. MACCEs included MACEs along with cerebrovascular disease, including cerebral hemorrhage and acute cerebral infarction. All patients were followed up for an average of 42 months through follow-up records from telephone calls or visits to the First Affiliated Hospital of Zhengzhou University.

### Statistical analysis

All data analyses were performed using the statistical software SPSS 26.0 for Windows (SPSS, Inc., Chicago, IL, USA). Continuous data are expressed as mean ± standard deviation. One-way analysis of variance (ANOVA) was employed to detect differences between groups when continuous variables exhibited both a normal distribution and a chi-square value, whereas the Kruskal–Wallis test was utilized when either of these conditions was not met. Categorical data are expressed as frequencies and percentages, and these variables were compared using the *χ*^2^ test. Kaplan–Meier analysis was applied to compare the cumulative incidence of long-term outcomes between groups. and the log-rank test was used to identify significant differences between groups. To construct Cox models, univariable models were used to identify possible predictive variables. Then variables that were significant (*p* < 0.05) in the univariable Cox model were entered simultaneously into the multivariable Cox model. Hazard ratios (HRs) and their 95% confidence intervals (CIs) were calculated. A two-sided *p* < 0.05 represented a significant difference.

## Results

### Baseline characteristics of postmenopausal CAD patients in the three SIRI groups

**Figure 1:**
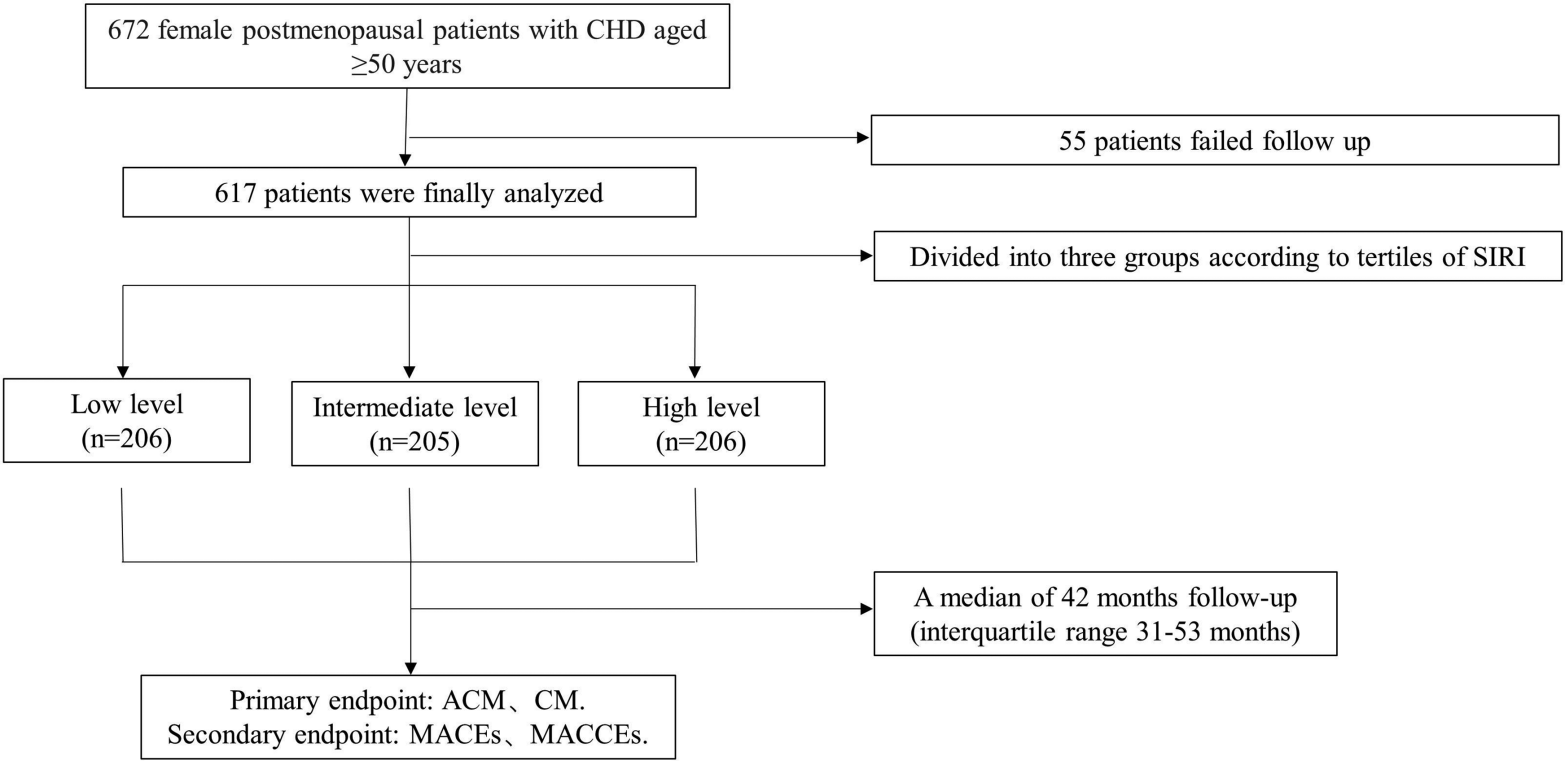
Study flowchart.

Initially, 672 older postmenopausal female patients with a diagnosis of CAD were reviewed, including 327 cases of ACS and 340 cases of CCS. Fifty-five patients were lost to follow-up because their contact information changed during the follow-up period, and these patients were excluded from this study. Finally, a total of 617 patients were included, including 297 cases of ACS and 320 cases of CCS. The patient flow chart is shown in Figure 1.

The 617 patients included in this study were divided into three groups according to SIRI tertiles, including the low level group (SIRI <0.73; *n* = 206), the intermediate level group (1.29≥ SIRI <0.73; *n* = 205), and the high level group (SIRI ≥1.29; *n* = 206). The baseline data for the patients in these three groups are presented in Table 1. The three groups showed statistically significant differences in age, white blood cell count, neutrophil count, monocyte count, lymphocyte count, platelet count, total triglycerides, HDL-C level, fibrinogen level, creatinine level, AST level, albumin level, fasting glucose level, glycosylated hemoglobin level, prevalence of diabetes mellitus, and prevalence of single-branch coronary artery lesions. No significant differences were found among the groups in the prevalence of hypertension, total cholesterol level, LDL-C level, or medication use. None of the patients had a history of smoking or alcohol consumption.

**Table 1:** Characteristics of postmenopausal CAD patients in the three groups divided by SIRI tertiles.

Variables	Low level SIRI	Intermediate level SIRI	High level SIRI	*p*-value
	(SIRI <0.73)	(1.29≥ SIRI <0.73)	(SIRI ≥1.29)	
SIRI				
Age, years	63.98 ± 8.27	65.60 ± 8.42	67.75 ± 8.91	<0.001
WBC (×10^9^/L)	5.22 ± 1.31	6.19 ± 1.35	8.31 ± 2.64	<0.001
Neut (×10^9^/L)	2.86 ± 0.87	3.84 ± 0.88	6.17 ± 2.55	<0.001
Mono (×10^9^/L)	0.34 ± 0.09	0.44 ± 0.11	0.55 ± 0.18	<0.001
Lymph (×10^9^/L)	1.87 ± 0.62	1.73 ± 0.56	1.47 ± 0.53	<0.001
Hb (g/L)	122.72 ± 14.69	123.13 ± 13.33	121.35 ± 14.36	0.414
PLT (×10^9^/L)	221.10 ± 60.98	225.54 ± 63.17	242.48 ± 73.79	0.003
TC (mmol/L)	4.17 ± 1.12	3.98 ± 0.96	4.02 ± 1.1	0.192
TG (mmol/L)	1.75 ± 1.43	1.84 ± 1.1	1.55 ± 0.88	0.038
HDL-C (mmol/L)	1.15 ± 0.28	1.09 ± 0.25	1.14 ± 0.27	0.048
LDL-C (mmol/L)	2.45 ± 0.97	2.31 ± 0.8	2.37 ± 0.95	0.281
Fib (g/L)	2.90 ± 0.52	3.15 ± 0.61	3.46 ± 0.98	<0.001
D-Dimer (mg/L)	0.25 ± 0.45	0.31 ± 0.68	0.45 ± 1.52	0.101
Cr (µmol/L)	63.09 ± 30.9	65.17 ± 20.04	70.88 ± 41.41	0.039
BUN (mmol/L)	5.52 ± 1.78	5.61 ± 1.91	6.84 ± 10.85	0.069
UA (µmol/L)	284.41 ± 81.81	295.62 ± 83.8	297.11 ± 99.59	0.285
ALT (U/L)	23.24 ± 21.29	28.74 ± 53.17	31.03 ± 43.07	0.149
AST (U/L)	29.06 ± 26.3	32.03 ± 43.07	53.95 ± 103.86	<0.001
TBIL (µmol/L)	9.14 ± 3.51	9.83 ± 5.41	10.06 ± 4.73	0.113
DBIL (µmol/L)	3.83 ± 1.6	4.13 ± 2.77	4.27 ± 2.23	0.128
IBIL (µmol/L)	5.25 ± 2.38	5.50 ± 2.94	5.75 ± 3.29	0.208
Alb (g/L)	41.89 ± 4.15	41.68 ± 3.42	40.07 ± 4.28	<0.001
FPG (mmol/L)	5.94 ± 2.5	6.40 ± 2.54	7.13 ± 3.57	<0.001
HbA1c (%)	6.43 ± 1.25	6.79 ± 1.32	6.70 ± 1.46	0.030
Hypertension (n,%)	138 (37.0)	147 (71.7)	147 (71.4)	0.508
Diabetes (n,%)	56 (27.2)	79 (38.5)	70 (34.0)	0.049
AF (n,%)	6 (2.9)	11 (5.4)	14 (5)	0.189
Number of lesions (n,%)				
1-VD	58 (28.2)	43 (21.0)	28 (13.6)	0.001
2-VD	53 (25.7)	49 (23.9)	62 (30.1)	0.344
3-VD	95 (46.1)	114 (55.6)	117 (56.8)	0.059
LM	13 (6.3)	14 (6.8)	19 (9.2)	0.487
Medication use (n,%)				
Antiplatelet drugs	206 (100)	205 (100)	206 (100)	1.000
Statins	206 (100)	205 (100)	206 (100)	1.000
*β*-blockers	68 (33.0)	57 (27.8)	50 (24.3)	0.141
ACEI or ARB	103 (50.0)	96 (46.8)	78 (37.9)	0.037

WBC, white blood cell; Neut, neutrophil; Mono, monocyte; Lymph, lymphocyte; Hb, hemoglobin; PLT, platelet; TG, triglycerides; TC, total cholesterol; HDL-C, high-density lipoprotein cholesterol; LDL-C, low-density lipoprotein cholesterol; Fib, fibrinogen; Cr, creatinine; BUN, blood urea nitrogen; UA, uric acid; ALT, alanine transaminase; AST, aspartate transaminase; TBIL, total bilirubin; DBIL, direct bilirubin; IBIL, indirect bilirubin; Alb, albumin; FPG, fasting blood glucose; HbA1c, glycosylated hemoglobin; AF, atrial fibrillation; LM, left main trunk lesion; ACEI, angiotensin-converting enzyme inhibitor; ARB, angiotonin receptor blocker.

### Comparison of the incidence of each outcome among the SIRI groups

Table 2 presents the comparison of the incidence of each outcome among the three SIRI groups. With 7 (3.4%) ACMs in the low level SIRI group and 19 (9.4%) ACMs in the high level SIRI group, the frequency of ACMs was significantly higher in the high level SIRI group (*p* = 0.014). The incidence of CM also was significantly higher in the high level SIRI group than in the low SIRI group (8.4% vs. 2.4%, *p* = 0.003). Furthermore, both MACEs (41.9% vs. 27.8%, *p* = 0.01) and MACCEs (45.3% vs. 32.2%, *p* = 0.024)occurred at a significantly higher frequency in the high level SIRI group compared with the low SIRI group.

**Table 2:** Outcome comparison among groups.

Outcomes	Low level SIRI (SIRI <0.73)	Intermediate level SIRI (1.29≥ SIRI <0.73)	High level SIRI (SIRI ≥1.29)	*p*-value
ACM (*n*, %)	7 (3.4)	8 (3.9)	19 (9.4)	0.014
CM (*n*, %)	5 (2.4)	5 (2.4)	17 (8.4)	0.003
MACEs (*n*, %)	57 (27.8)	67 (32.5)	85 (41.9)	0.01
MACCEs (*n*, %)	66 (32.2)	82 (39.8)	92 (45.3)	0.024

*p*-values represent comparisons among three groups.

ACM, all-cause mortality, CM, cardiac mortality, MACEs, major adverse cardiac events, MACCEs, major adverse cardiovascular and cerebrovascular events.

### Univariable and multivariable Cox regression analyses of predictive factors for each outcome

To identify independent predictors of each outcome in older menopausal women with CAD, we conducted Cox proportional hazard analyses. The results are shown in Table 3. All potential confounding variables were initially incorporated into a univariate Cox analysis. Subsequently, significant variables (*p* < 0.05) were included in multivariable Cox regression modeling. From the univariable analysis, the incidence rates of ACM, CM, MACEs, and MACCEs were significantly higher in the high level SIRI group than in the low level SIRI group [ACM: HR = 3.214 (95% CI: 1.350–7.649), *p* = 0.008; CM: HR = 3.979 (95% CI: 1.647–10.792), *p* = 0.007; MACE: HR = 1.752 (95% CI: 1.252–2.451), *p* = 0.001; MACCE: HR = 1.650 (95% CI: 1.203–2.264), *p* = 0.002]. From the multivariable Cox regression analysis, compared with patients in the low level SIRI group, those in the high level SIRI group had a 2.249-fold greater risk of developing ACM (HR = 2.581, 95% CI:1.045–6.373, *p* = 0.040), a 2.297-fold greater risk of developing CM (HR = 2.798, 95% CI: 0.972–8.060, *p* = 0.057), a 0.672-fold greater risk of developing MACEs (HR = 1.623, 95% CI: 1.123–2.346, *p* = 0.01), and a 0.585-fold greater risk of developing MACCEs (HR = 1.558, 95% CI: 1.100–2.207, *p* = 0.012).

**Table 3:** Univariable and multivariable Cox regression analyses of factors predictive of outcomes.

Outcomes	Non-adjusted univariable		Adjusted multivariable	
	HR (95% CI)	*p*-value	HR (95% CI)	p-value
ACM				
SIRI tertile				
1, Low	1		1	
2, Intermediate	1.213 (0.440–3.347)	0.709	1.033 (0.357–2.992)	0.952
3, High	3.214 (1.350–7.649)	0.008	2.581 (1.045–6.373）	0.04
FPG (mmol/L)	1.117 (1.032–1.210)	0.006	–	–
WBC (×109/L)	1.202 (1.070–1.351)	0.002	–	–
Hb (g/L)	0.980 (0.957–0.994)	0.006	–	–
Cr (µmol/L)	1.006 (1.002–1.011)	0.003	1.007 (1.002–1.012)	0.011
UA (µmol/L)	1.005 (1.002–1.009)	0.001	1.004 (1.001–1.008)	0.007
CM				
SIRI tertile				
1, Low	1		1	
2, Intermediate	1.062 (0.307–3.668)	0.924	1.007 (0.286–3.542)	0.991
3, High	3.979 (1.647–10.792)	0.007	2.798 (0.972–8.060)	0.057
FPG (mmol/L)	1.140 (1.053–1.234)	0.001	1.083 (1.001–1.171)	0.048
WBC (×109/L)	1.235 (1.100–1.386)	<0.001	–	–
Hb (g/L)	0.979 (0.965–0.994)	0.006	–	–
Cr (µmol/L)	1.007 (1.002–1.011)	0.006	1.007 (1.000–1.013)	0.044
UA (µmol/L)	1.005 (1.001–1.009)	0.006	1.003 (1.000–1.007)	0.073
MACEs				
SIRI tertile				
1, Low	1		1	
2, Intermediate	1.238 (0.869–1.762)	0.237	1.159 (0.790–1.702)	0.45
3, High	1.752 (1.252–2.451)	0.001	1.623 (1.123–2.346)	0.01
FPG (mmol/L)	1.068 (1.027–1.111)	0.001	–	–
WBC (×109/L)	1.112 (1.050–1.178)	<0.001	–	–
Fib (g/L)	1.257 (1.049–1.507)	0.013	–	–
D-Dimer (mg/L)	1.189 (1.106–1.279)	<0.001	1.169 (1.086–1.257)	<0.001
UA (µmol/L)	1.002 (1.001–1.004)	0.003	1.002 (1.000–1.004)	0.014
HbA1c (%)	1.134 (1.033–1.246)	0.009	1.138 (1.030–1.258)	0.011
Alb (g/L)	0.946 (0.914–0.979)	0.002	–	–
MACCEs				
SIRI tertile				
1, Low	1		1	
2, Intermediate	1.312 (0.949–1.815)	0.1	1.231 (0.864–1.755)	0.25
3, High	1.650 (1.203–2.264)	0.002	1.558 (1.100–2.207)	0.012
FPG (mmol/L)	1.074 (1.035–1.114)	<0.001	–	–
WBC (×109/L)	1.079 (1.020–1.142)	0.008	–	–
D-Dimer (mg/L)	1.184 (1.099–1.276)	<0.001	1.172 (1.089–1.261)	<0.001
UA (µmol/L)	1.002 (1.001–1.003)	0.007	1.002 (1.000–1.003)	0.026
HbA1c (%)	1.142 (1.047–1.247)	0.003	1.148 (1.046–1.259)	0.004
Alb (g/L)	0.949 (0.918–0.980)	0.002	–	–

### Kaplan–Meier survival analysis

**Figure 2:**
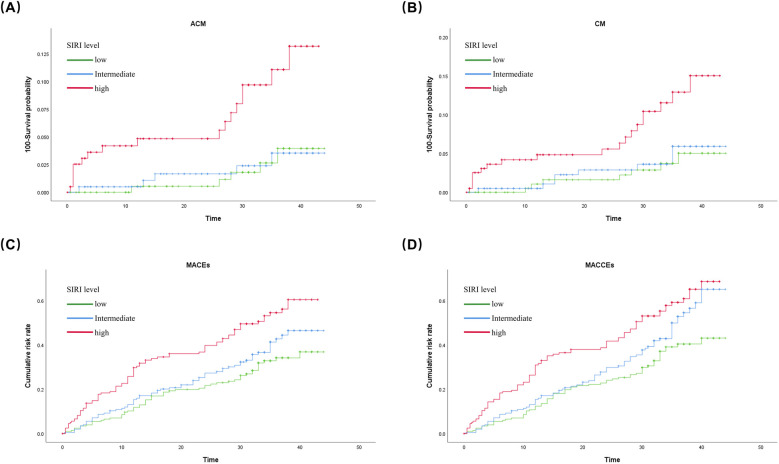
Cumulative Kaplan-Meier estimates of the time to the first adjudicated occurrence of **(A)** ACM; **(B)** CM; **(C)** MACEs; and **(D)** MACCEs.

The Kaplan–Meier survival analysis revealed that patients in the high level SIRI group had greater risks of CM (log rank, *p* = 0.005), ACM (log rank, *p* = 0.001), MACEs (log rank, *p* = 0.003), and MACCEs (log rank, *p* = 0.005) than those in the low level SIRI group. The results are shown in Figure 2.

## Discussion

The present study investigated the predictive value of the novel inflammatory marker SIRI for adverse prognoses in postmenopausal women with CAD aged 50 years or older. The results showed that the SIRI could significantly predict several adverse outcomes in this population. In particular, the SIRI had a high predictive value for the risk of death. Cox regression modeling showed that the risk of ACM was 1.581-fold higher in the high level (third tertile) SIRI group compared with the low level (first tertile) SIRI group (HR = 2.581, 95% CI:1.045–6.373, *p* = 0.040), and the risk of cardiogenic mortality was 1.798-fold higher in the high level SIRI group compared with the low level SIRI group (HR = 2.798, 95% CI: 0.972–8.060, *p* = 0.057). In addition, the risks of MACEs and MACCEs were 62.3% higher (HR = 1.623, 95% CI: 1.123–2.346, *p* = 0.01) and 55.8% higher (HR = 1.558, 95% CI: 1.100–2.207, *p* = 0.012), respectively, in the high level SIRI group compared with the low level SIRI group. The Kaplan–Meier survival curves also showed a gradually shorter survival time and a higher risk of adverse cardiovascular events with increasing SIRI values.

In females, estrogen is known to have anti-inflammatory effects, via downregulation of nuclear factor kappa B (NF-*κ*B), interleukin-1 (IL-1), and IL-6 and the promotion of anti-inflammatory M2 macrophages, T regulatory cells (Tregs), IL-4 expression, IL-10 expression, and transforming growth factor beta (TGF-*β*) expression. Estrogen also contributes to vasodilatation of the vascular endothelium, blockade of hyperglycemia-induced smooth muscle cell proliferation, and promotion of endothelial repair through estrogen receptor alpha (ER*α*). Through these combined effects, estrogen has a cardiovascular protective effect in women (18, 19). In postmenopausal women after the age of 50 years, the decline in estrogen leads to increases in innate immune activation and pro-inflammatory cytokines, while aging also results in a state of chronic low-grade inflammation, with increased levels of inflammatory cytokines, such as IL-6 and tumor necrosis factor alpha (TNF-α) (20, 21). Accordingly, the risk of atherosclerosis is 3.4-fold higher in postmenopausal women than in premenopausal women (22). In the pathogenesis of ACS, whereas premenopausal women tend to exhibit thick fibrous caps and small necrotic core plaques that undergo erosion, postmenopausal women are more prone to experience rupture of large necrotic core plaques with thin fibrous caps (23). Histologic studies have shown that plaque rupture is highly correlated with inflammation, whereas no increase in inflammation is found during plaque erosion (24). The level of CRP, the classic inflammatory factor in peripheral blood, is significantly elevated in postmenopausal women (25), and a high level of CRP is associated with the occurrence of cardiovascular events (coronary heart disease-related death, nonfatal myocardial infarction or stroke, and coronary revascularization surgery) in postmenopausal women (12). Elevated CRP levels have also been observed in patients with depression, which is another a high risk factor for coronary heart disease in women (26). In summary, elevated levels of systemic inflammation in postmenopausal women are associated with the development of CAD and adverse cardiovascular events. The peripheral blood leukocyte level offers a convenient, inexpensive, and readily available indicator of inflammation. Leukocyte counts and neutrophil counts have been shown to be associated with cardiovascular events in postmenopausal women (27, 28). However, studies investigating the predictive value of derived inflammatory markers for the prognosis of postmenopausal women with CAD are lacking. In the present study, we demonstrate the good predictive value of SIRI for prognosis in postmenopausal women with CAD.

CAD is a chronic inflammatory disease, and a variety of inflammatory immune cells in the peripheral blood are involved in the formation of atherosclerotic plaques (29). The SIRI represents the combined level and interbalance of monocytes, neutrophils, and lymphocytes. Mononuclear macrophages are well established as the major immune cells in atherogenesis and are involved in the entire disease process, including the onset and progression of atherosclerosis, formation of unstable plaques, plaque rupture, intra-plaque vascularization, and post-infarction myocardial remodeling (30). Monocytes adhere and migrate to the subendothelium of arteries through chemokine attraction (mainly CCL2) during the stimulation of inflammation. Once there, they further differentiate into macrophages and then phagocytose lipids to form foam cells, which activate NF-*κ*B targets to sustain the inflammatory response and further promote the formation of large numbers of foam cells to form the lipid core of plaques (31, 32). The monocyte count was shown to predict new development of atherosclerotic plaques and to be an independent predictor of prognosis in CAD (33, 34). Neutrophils act as fast reactive inflammatory cells. A previous study showed that blocking the CXCL12/CXCR4 chemokine receptor axis in mice can accelerate the process of atherosclerosis by causing an increase in the number of neutrophils in peripheral blood and plaques (35). Due to the short half-life of neutrophils in peripheral blood, the role of neutrophils in human atherosclerotic formation needs to be determined in further studies. However, clinical studies have shown that neutrophil counts correlate with the severity of CAD as well as the prognosis of patients with ACS and patients after percutaneous coronary intervention (PCI) (36, 37). Different lymphocyte subsets have different functions in atherosclerosis, with T helper 1 (Th1) cells having pro-atherogenic effects and Th2 cells, Tregs and B cells having anti-atherogenic effects (38). Overall, lymphocytes are protective and regulatory factors in atherosclerosis. In clinical studies, lymphocyte counts have been shown to correlate with poor prognosis in patients with CAD in absolute terms and to have diagnostic value in acute myocardial infarction (AMI) (39).

Peripheral blood immune cells do not exist independently, and CAD progression requires the participation of all types of immune cells and their combined effects. The novel inflammatory marker SIRI was originally developed for cancer risk prediction (40). However, this marker has also been widely used for risk prediction in CAD. Two large population-based prospective cohort studies have shown that an elevated SIRI correlates with an increased incidence of all-cause cardiovascular death, cardiac death, stroke (hemorrhagic and ischemic), and AMI (17, 41). A single-center prospective study evaluated the predictive value of the MLR, NLR, PLR, SII, and SIRI for the occurrence of MACEs (nonfatal myocardial infarction, nonfatal ischemic stroke, and all-cause death) in patients undergoing PCI for ACS. Although all inflammatory markers were found to correlate with the occurrence of MACEs, the predictive value of the SIRI was superior to that of the other markers (42). The SIRI has a higher predictive value than SII in predicting the occurrence of postoperative adverse cardiovascular events in patients with AMI (area under the curve = 0.678 for SII and 0.707 for SIRI in the predictive value for MACE) (43). The SIRI also has predictive value for poor prognosis in non-ST-segment elevation myocardial infarction (44). A study assessing the predictive value of the novel inflammatory markers SII, SIRI, and AISI for mortality after noncorporeal coronary artery bypass grafting in patients with CAD found that only the SIRI had a good predictive value after correction by multifactorial regression analysis (45). The SIRI has a higher sensitivity for risk prediction of the occurrence of adverse events related to CAD than other derived inflammatory indicators. In conclusion, a multitude of studies have substantiated that SIRI is linked to the incidence of adverse cardiovascular events in patients with varying degrees of CAD. Nevertheless, no study had yet investigated the predictive value of SIRI for the prognosis of patients with CAD in different age and gender subgroups. In the present study, other derived inflammatory indicators, including the MLR, NLR, PLR, and SII, did not have good predictive value for poor prognosis of CAD in postmenopausal women aged 50 years and older, whereas the SIRI did. A recent study indicate that elevated SIRI values are significantly associated with an increased risk of stroke and its subtypes in elderly patients with hypertension (46). For postmenopausal women as a specific study population, recent studies have found that an elevated SIRI is associated with cardiovascular death in postmenopausal women with osteoporosis or reduced bone mass (47, 48). This may be due to the involvement of multiple immune cells and inflammatory mediators in the pathogenesis of osteoporosis and atherosclerosis (49). In a study of the association between derived inflammatory indicators and the occurrence of ACS in 250 elderly women, the SIRI was found to be significantly higher in patients with ACS but was not included in the construction of logistic regression models after elimination of covariates by backward stepwise regression analysis (50). It is evident that a correlation exists between the SIRI and a wide range of diseases affecting the cardiovascular system, as well as other systemic diseases. Furthermore, the SIRI is an excellent indicator of the level of systemic inflammation.

The present study demonstrates for the first time that the novel derived inflammatory index SIRI has good predictive value for the assessment of multiple adverse outcomes in postmenopausal women with CAD. Thus, the SIRI is anticipated to serve as a pivotal reference index and scoring program for prognostic assessment of clinical postmenopausal women with coronary artery disease. Some limitations of the study should be noted. First, this study was a single-center retrospective cohort study with limited sample size, and thus, the results need to be further validated in a multicenter study with a large sample set and, ultimately, by meta-analysis in the future. Furthermore, despite our efforts to mitigate confounding factors through a sufficient sample size and rigorous statistical techniques, retrospective studies are inherently constrained by limitations such as selection bias, information bias, recall bias, and forgetting bias. Also, in this study, patients were not further divided into subgroups according to different clinical conditions/comorbidities to analyze the prognostic value of SIRI. In the future, a larger sample size is needed to explore the predictive value of SIRI for the prognosis of postmenopausal patients with ACS, CCS, and those suffering from comorbidities.

## Conclusion

In conclusion, our study demonstrates that SIRI is an independent predictor of poor prognosis in postmenopausal women with CAD, including CM, ACM, MACEs, and MACCEs. Accordingly, the SIRI can be used in strategies to identify high-risk groups among postmenopausal women with CAD.

## Data Availability

The original contributions presented in the study are included in the article/Supplementary Material, further inquiries can be directed to the corresponding author.
